# Relationship between triglyceride-glucose index and gallstones risk: a population-based study

**DOI:** 10.3389/fendo.2024.1420999

**Published:** 2024-07-11

**Authors:** Quanhui Liao, Yongtai Chen, Quanshui Peng, Chunying Li

**Affiliations:** Department of Hepatobiliary Surgery, Longyan First Hospital Affiliated to Fujian Medical University, Longyan, China

**Keywords:** Triglyceride-glucose index, gallstones, National Health and Nutrition Examination Survey, risk, insulin resistance

## Abstract

**Background:**

Globally, gallstones represented a prevalent condition of the digestive system, heavily affected by metabolic dysfunctions such as obesity, dyslipidemia, insulin resistance, and diabetes. The triglyceride-glucose (TyG) index served as an accessible novel indicator for evaluating insulin resistance, offering a precise reflection of metabolic conditions. However, no studies have yet explored their relationship. The link between the TyG and gallstone risk was the primary purpose of this study.

**Methods:**

Utilized data from the public database, the National Health and Nutrition Examination Survey, for the years 2017-2020. The logit model was utilized to elucidate the connection between the TyG and the gallstones risk. The restricted cubic spline (RCS) analysis served to verify any non-linear relationships existing between them. Sensitivity analyses, encompassing both stratified and interaction analyses, were conducted to identify populations of particular interest and assess potential interactions between covariates and the TyG index.

**Results:**

A total of 4544 individuals were included. The risk of gallstones in high group was 1.6 times that of the low group. The potential cut-off value for the TyG index was 6.19. Above this threshold, there was a 40% heightened risk of gallstones with each one-unit increment in the TyG. The RCS analysis revealed the absence of a non-linear association between them. The populations warranting particular focus included those over 60 years, non-White people, individuals with a body mass index ≥25, smokers, drinkers, those with hypertension, and diabetes. Apart from smoking history, alcohol consumption, and history of diabetes, there were no interactions between other variables and the TyG index.

**Conclusion:**

The current study represented the inaugural investigation into the link between TyG index and the risk of gallstones. A positive correlation existed between them, signifying that an increase in TyG paralleled an elevated risk of gallstones. No non-linear relationship has been found between them. Besides, a 40% increase in gallstone risk accompanied each unit rise in TyG. Considering the convenience and accessibility of TyG in clinical settings, it has a promising potential for clinical application.

## Introduction

Gallstones were a widespread chronic disease of the digestive system found globally. The worldwide incidence of gallstones in adults ranged from 10 to 20% (1). Gallstones were primarily categorized as cholesterol stones, pigment stones, and mixed stones, where cholesterol and cholesterol-dominated mixed stones accounted for more than 80% of all stones (2). While typically asymptomatic, around 20% of individuals with gallstones would show symptoms including biliary pain or infections during their lifetime, with 1-2% facing severe complications. Such complications could include acute pancreatitis, acute obstructive purulent cholangitis, and gallbladder cancer (3, 4).

Earlier studies have identified demographic factors like age, gender, and race as risk factors for gallstones. The occurrence of gallstones grew with age, peaking after 50 for females and 60 for males. Females have a substantially higher risk of developing gallstones than males. Additionally, the prevalence of gallstones was elevated among Hispanic populations in Central and South America, average in Asians, and lowest among Africans (1, 5–7). Recent studies found that the development of cholesterol gallstones was deeply affected by metabolic disorders. Factors such as obesity, lipid abnormalities, insulin resistance, and diabetes contributed to this condition. For example, with every 5 unit increased in body mass index (BMI) or presence of diabetes, the risk of developing gallstones was 1.63 and 1.56 respectively (8, 9). Both insulin resistance and diabetes were distinct risk factors for the development of gallstones (10, 11). Although metabolic surgery, such as Roux-en-Y gastric-bypass lead to improvement of glycemic control and consequently cholelilthiasis reduction, the abrupt weight reduction has an opposite effect predisposing to gallbladder stones formation (12). In contrast to the less changeable demographic features, metabolic factors could be more easily altered through changes in one’s lifestyle.

The triglyceride-glucose (TyG) index was an accessible marker that merged fasting triglycerides and glucose for assessing insulin resistance (13). It accurately reflected an individual’s metabolic status. Relative to traditional insulin resistance markers like the intravenous glucose tolerance test, which was expensive and invasive (14), and the homeostasis model assessment of insulin resistance that has not yet gained broad clinical application (15), the TyG index was more accessible and cost-efficient. Prior research demonstrated a notable connection between the TyG and diseases like diabetes, cardiovascular disorders, and metabolic disorders (16–19). Considering that metabolic abnormalities profoundly affected gallstones, there might be an underlying link between the TyG and gallstones.

The main objective of the current research was to assess the connection between them according to a large dataset, the National Health and Nutrition Examination Survey (NHANES), encompassing the effect of continuous variations in the TyG index. Based on this, the study preliminarily identified the populations that required focused attention. This was intended to enable a more convenient and accurate evaluation of individual gallstone risks.

## Methods

### Study population

**Figure 1:**
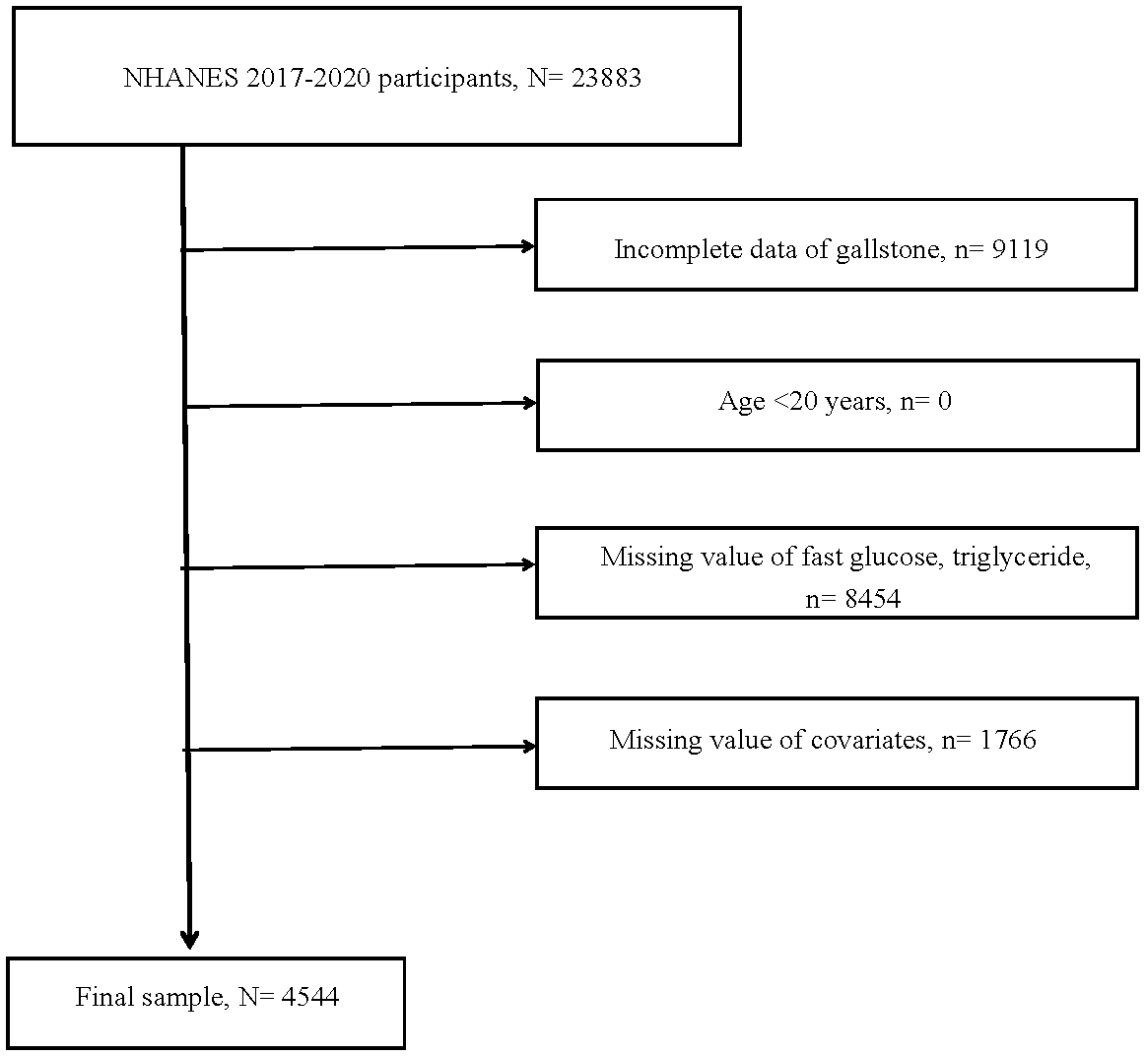
Flow chart of study. N, the number of individuals being included. n, the number of individuals being excluded.

NHANES was a comprehensive, ongoing survey involving large samples, collecting clinical measurements, laboratory data, and questionnaire information to detail public health, nutrition, diseases, and risk factors. The process for inclusion and exclusion in the current study was thoroughly outlined in **Figure 1**. From the NHANES database, we selected the cohort from 2017 to 2020, comprising 23883 participants. Due to unclear information about gallstones, 9119 participants were excluded. A total of 8454 individuals were excluded because they lacked data on fasting triglycerides and glucose. 1766 individuals were excluded due to missing data on alanine aminotransferase (ALT), total cholesterol (TC), BMI, gender, age, race, smoking history, alcohol consumption, history of hypertension, or diabetes. In the end, 4544 individuals were included in the study. All participants were older than 20 years.

### Identification of gallstones

Gallstones were determined based on the results of a standardized questionnaire. In other word, if a participant answered “yes” to the question “Have you ever been told you have gallstones?”, they were considered to have gallstones.

### TyG index

The TyG index, which was made up of fasting triglycerides and glucose, was calculated using the formula: Ln [fasting triglycerides (mg/dL) × fasting glucose (mg/dL)/2]. Participants were classified into three groups, each approximately comprising one-third of the total, according to the TyG index levels. Specially, low (6.19≤TyG ≤ 8.15, n=1515), moderate (8.15< TyG ≤ 8.75, n=1511), and high (8.75< TyG ≤ 11.94, n=1518).

### Covariates

From the NHANES database, we pulled a number of variables that could be linked to gallstone formation, encompassing history of hypertension or diabetes, smoking or alcohol status, ALT, and TC, BMI, gender, age, race. The definitions and categorizations of these variables were displayed in the **Supplementary Methods**.

### Statistical analysis

This research was carried out in strict accordance with NHANES recommended practices. All analyses were based on weighted data. The Wilcoxon rank-sum test was employed to discern differences among continuous variables. The chi-squared test clarified differences among categorical variables. Multivariate logit analysis, a frequently used statistical method, was applied to analyze the connection between the TyG and the gallstones risk. Specifically, crude model, model1, and model2 were three crucial models in this research. The first model solely incorporated the TyG index. The second model added demographic characteristics such as age, gender, and race as adjusting factors. The last and most significant model not only included age, gender, and race but also incorporated BMI, smoking and drinking status, hypertension, diabetes, ALT, and TC for comprehensive adjustment. A noteworthy point was the exploration of a potential non-linear association between the TyG and gallstone risk in this study. The non-linear relationship was investigated using restricted cubic spline (RCS) analysis. Should a non-linear correlation be present, the inflection points would be further defined (using recursive algorithms), and threshold effects would be assessed (using segmented logit regression models). Moreover, to assist doctors in clinical settings in more effectively evaluating the dynamic effects of continuous changes in the TyG index on gallstone risk, additional analyses were conducted. The TyG index served as continuous variable for following analysis, without grouping based on its levels, to evaluate the effect of each unit change on gallstone risk. Sensitivity analysis, incorporating stratified analysis and interaction analysis, was employed to increase the robustness of this research. Stratified analyses, formulating based on factors such as age, gender, race, BMI, smoking and drinking habits, hypertension, diabetes, as well as ALT and TC levels. BMI was segmented into ≥25 and <25 categories. ALT was split into >40 and ≤40 groups. TC was categorized into >5.18 and ≤5.18 groups. From the stratified analysis, it was possible to identify dominant subgroups. Interaction analysis was employed to evaluate potential interactions among different covariates with the TyG index. All statistical analyses were completed using R4.3.1. When *P*-value was less than 0.05, it indicated statistical significance.

## Results

### Baseline characteristics

In total, 4544 individuals who met the criteria were incorporated into this study for further analysis. Participants were divided into low, moderate, and high groups, with 1515, 1511, and 1518 individuals respectively. The median TyG index values were 7.86, 8.46, and 9.12, while the mean values were 7.78, 8.45, and 9.24, respectively. Distinct characteristics were observed in the moderate and high groups compared to the low group. On one hand, some values or proportions were higher, including age, average values of TyG index, BMI, TC, and ALT, as well as the proportions of males, smokers, hypertension, and diabetes. On the other hand, the proportion of drinkers in the moderate and high groups was lower. There were no racial differences among the three groups (**Table 1**).

**Table 1:** Baseline characteristics of patients with gallstone in the NHANES 2017–2020 cohort.

Characteristics	TyG				
	Total	Low group 7.86 [6.19,8.15]	Moderate group 8.46 (8.15,8.75]	High group 9.12 (8.75,11.94]	*P*
Participants, n	4544	1515	1511	1518	
TyG, mean	8.47(8.44,8.50)	7.78(7.76,7.80)	8.45(8.43,8.46)	9.24(9.20,9.28)	< 0.0001
Age, year	46.23 (45.18,47.29)	41.12 (39.59,42.65)	47.48 (46.28,48.68)	50.52 (49.16,51.89)	< 0.0001
Gender, n (%)					< 0.001
Male	2247(50.85)	627(42.70)	772(53.46)	848(57.05)	
Female	2297(49.15)	888(57.30)	739(46.54)	670(42.95)	
Race, n (%)					0.48
White people	1539(64.04)	474(63.03)	522(65.43)	543(63.72)	
Non-White people	3005(35.96)	1041(36.97)	989(34.57)	975(36.28)	
BMI, Kg/m2	29.42 (29.00,29.83)	26.35 (25.77,26.93)	30.45 (29.83,31.07)	31.70 (31.19,32.22)	< 0.0001
ALT, U/L	23.11 (22.26,23.96)	18.79 (17.36,20.22)	23.99 (22.55,25.43)	26.92 (25.86,27.98)	< 0.0001
TC, mmol/L	4.81(4.75,4.88)	4.40(4.31,4.50)	4.88(4.80,4.95)	5.20(5.10,5.30)	< 0.0001
Smoke status, n (%)					0.001
No/Former	3677(83.43)	1256(86.59)	1211(83.83)	1210(79.57)	
Yes	867(16.57)	259(13.41)	300(16.17)	308(20.43)	
Alcohol, n (%)					0.03
No/Former	515(7.53)	164(5.92)	166(7.84)	185(8.97)	
Yes	4029(92.47)	1351(94.08)	1345(92.16)	1333(91.03)	
Hypertension, n (%)					< 0.0001
No	2808(67.14)	1135(80.49)	908(65.34)	765(54.43)	
Yes	1736(32.86)	380(19.51)	603(34.66)	753(45.57)	
Diabetes, n (%)					< 0.0001
No	3639(86.33)	1421(96.90)	1299(90.02)	919(70.98)	
Yes	905(13.67)	94(3.10)	212(9.98)	599(29.02)	
Gallstone, n (%)					< 0.001
No	4127(90.27)	1424(92.60)	1374(91.91)	1329(86.04)	
Yes	417(9.73)	91(7.40)	137(8.09)	189(13.96)	

TyG, triglyceride-glucose; BMI, body mass index; ALT, alanine aminotransferase; TC, total cholesterol.

### Multivariate logit analysis

Across the crude model to model2, the TyG index high group showed a significantly increased risk of gallstones, with odds ratio (OR) values being 2.03 (*P*_trend_ *<*0.001), 2.06 (*P*_trend_ *<*0.001), and 1.60 (*P*_trend_ = 0.04) respectively. When the TyG was incorporated as a continuous variable in multivariate adjusted logit regression analysis, results implied that each unit increase in the TyG index raised the risk of gallstones by 44%, 57%, and 40%, respectively. Regardless of whether the TyG index was considered a categorical or continuous variable, the risk of gallstones increased with rising levels of the index (**Table 2**).

**Table 2:** Relationships of TyG with risk of gallstone from the NHANES 2017–2020 cohort.

TyG	OR, 95%CI		
	Crude Model	Model 1	Model 2
Low group	ref	ref	ref
Moderate group	1.10(0.73,1.65)	1.08(0.72,1.61)	0.86(0.54,1.37)
High group	2.03(1.40,2.95)	2.06(1.41,3.00)	1.60(1.01,2.61)
Per 1 U increment	1.44(1.22,1.70)	1.57(1.32,1.86)	1.40(1.11,1.78)
***P* for trend**	<0.001	0.001	0.003

TyG, triglyceride-glucose; BMI, body mass index; ALT, alanine aminotransferase; TC, total cholesterol.

Model 1: adjusted for age, gender, and race. Model 2: model 1+ adjusted for BMI, smoke and alcohol status, hypertension, diabetes, ALT, and TC.

### Non-linear relationships

**Figure 2:**
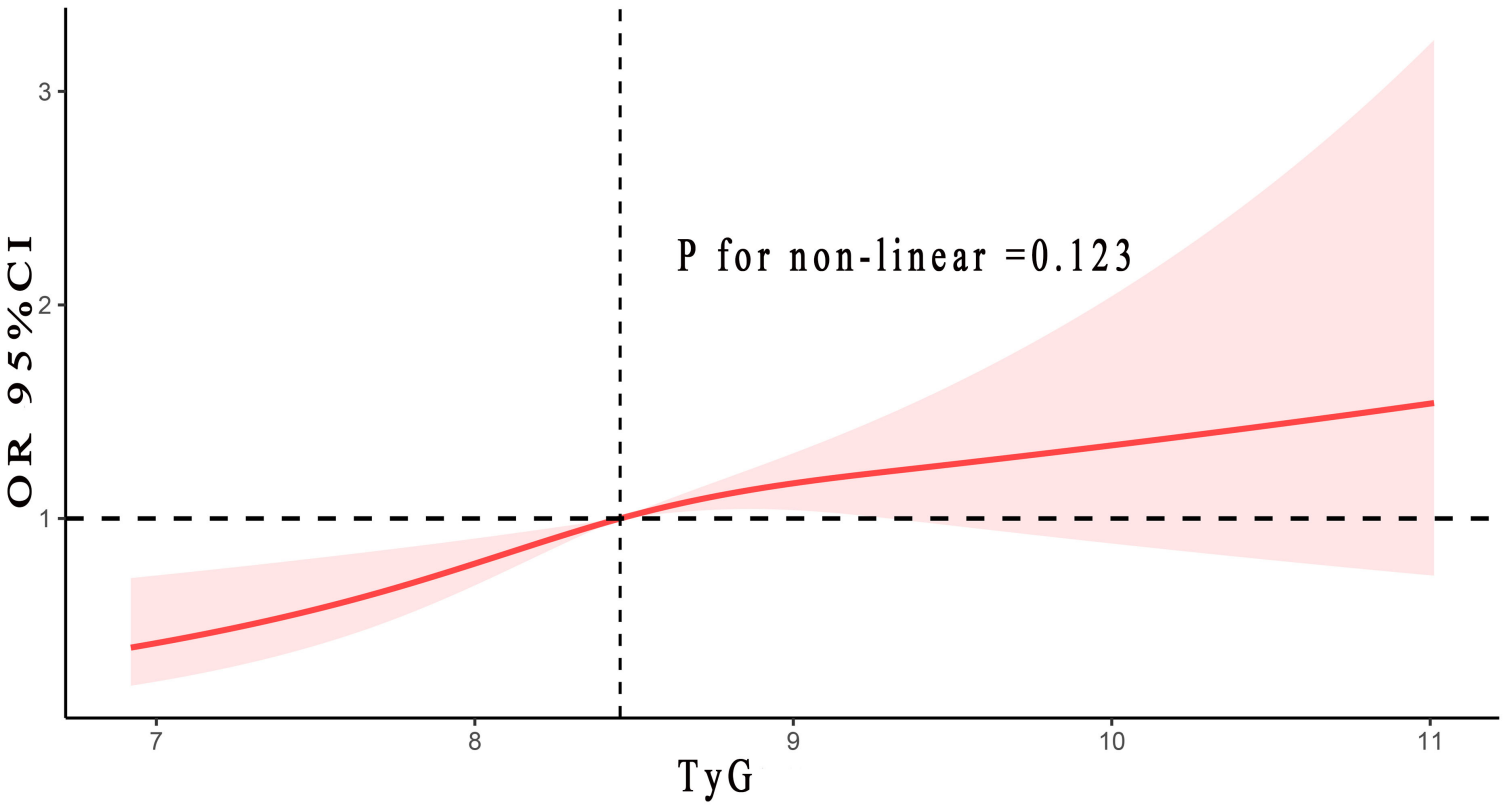
The correlation between TyG index and gallstones risk. Adjusted for age, gender, race, BMI, smoke and alcohol status, hypertension, diabetes, ALT and TC. TyG, triglyceride-glucose; BMI, body mass index; ALT, alanine aminotransferase; TC, total cholesterol.

According to **Figure 2**, non-linear association was not be found between the TyG and the risk of gallstones (*P*_non-linear_ = 0.123). With the rise of the TyG index, gallstones risk initially elevated significantly, then the rate of increase moderately slowed (yet continued to rise). In general, the rise of the TyG index corresponded to a heightened risk of gallstones.

### Sensitivity analysis

The results of stratified analysis indicated that the following populations need particular focus: those aged over 60, non-White people, individuals with a BMI of ≥25, smokers, drinkers, and people with hypertension or diabetes. According to the interaction analysis, there were no interactions between the TyG index and any variables except for smoking and alcohol status, and diabetes history (**Table 3**).

**Table 3:** Stratified analyses of the relationships between TyG and risk of gallstone from the NHANES 2017–2020 cohort.

Characteristics	TyG				
	Low group 7.86 [6.19,8.15]	Moderate group 8.46 (8.15,8.75]	High group 9.12 (8.75,11.94]	*P* for trend	*P* for interaction
Participants, n	1515	1511	1518		
Age					0.48
≤60	ref	1.11(0.57,2.14)	1.58(0.87,2.88)	0.11	
>60	ref	0.73(0.34,1.56)	1.99(1.09,3.65)	0.01	
Gender					0.72
Male	ref	1.15(0.57, 2.33)	2.02(0.89, 4.63)	0.08	
Female	ref	0.87(0.47,1.59)	1.62(0.89,2.96)	0.08	
Race					0.11
White people	ref	0.77(0.46,1.29)	1.53(0.86,2.74)	0.11	
Non-White people	ref	1.61(0.94,2.76)	2.56(1.35,4.83)	0.005	
BMI					0.22
<25	ref	0.68(0.29, 1.63)	0.83(0.36, 1.90)	0.43	
≥25	ref	1.12(0.61,2.05)	2.18(1.22,3.90)	0.01	
Smoke status					0.01
No/former	ref	0.78(0.48,1.25)	1.43(0.92,2.23)	0.08	
Yes	ref	4.67(1.54,14.21)	8.19(1.99,33.65)	0.005	
Alcohol					<0.001
No/former	ref	3.46(0.82, 14.64)	1.55(0.29, 8.38)	0.62	
Yes	ref	0.82(0.51,1.33)	1.72(1.08,2.73)	0.01	
Hypertension					0.05
No	ref	1.22(0.79,1.89)	1.54(0.91,2.61)	0.1	
Yes	ref	0.66(0.34, 1.31)	2.05(1.04, 4.04)	0.01	
diabetes					0.04
No	ref	0.84(0.53,1.33)	1.59(0.95,2.66)	0.07	
Yes	ref	7.65(2.13,27.49)	11.04(3.59,33.91)	0.005	
ALT					0.40
≤40	ref	0.87(0.53,1.45)	1.71(1.03,2.82)	0.03	
>40	ref	2.25(0.42,12.18)	3.69(0.76,17.80)	0.18	
TC					0.99
≤5.18	ref	0.92(0.59,1.44)	1.67(0.97,2.89)	0.07	
>5.18	ref	1.09(0.33,3.64)	2.14(0.67,6.79)	0.08	

TyG, triglyceride-glucose; BMI, body mass index; ALT, alanine aminotransferase; TC, total cholesterol.

## Discussion

For the first time, this study demonstrated the relationship between the TyG index and gallstones, derived from a publicly available, representative large sample cohort. Finding indicated that a positive association between them, showing a 60% increase in gallstone risk with the rise of the TyG index. Furthermore, the potential critical threshold value for the TyG index was 6.19. Above this threshold, each unit increase in the TyG index resulted in a 40% higher risk of developing gallstones. For the first time, this study found that there was no non-linear relationship between them. Subgroup analysis suggested that populations needing particular attention included those aged over 60, non-White people, individuals with a BMI of ≥25, smokers, drinkers, and those with hypertension or diabetes. Interaction analysis results showed that, except for smoking and alcohol status, and diabetes history, there were no interactions between other variables and the TyG index, indicating that the study results were stable and reliable.

The formation of gallstones was triggered by elevated levels of cholesterol or bilirubin in the bile (20). Depending on their chemical composition and appearance, gallstones were mainly divided into two types: cholesterol gallstones and pigment gallstones. Of these, cholesterol gallstones are more common. Formation of cholesterol gallstones was caused by a failure in maintaining cholesterol homeostasis within the bile, with the key factor being the disturbance of physicochemical balance of cholesterol solubility (21). several factors, such as hepatic cholesterol hypersecretion, facilitating the crystallization of cholesterol and gallstone formation (1). Increasing research found that the formation of cholesterol gallstones was profoundly affected by metabolic abnormalities, incorporating obesity, abnormal blood lipids, insulin resistance, and diabetes. Insulin resistance and diabetes stood as independent risk factors for gallstone formation (10, 11). Gallstones became symptomatic in around 20%, presenting from biliary colic pain to the more complicated gallstone ileus, demonstrating the need for immediate diagnosis with convenient applied indices, such as TyG (22).

Insulin resistance, defined as diminished sensitivity and reactivity to insulin, was a key indicator of type 2 diabetes mellitus. Composed of fasting triglycerides and fasting blood glucose, the TyG index served as a comprehensive, novel index for the convenient assessment of insulin resistance, proving to be a superior alternative to the homeostasis model assessment of insulin resistance index (23, 24). Apart from diabetes, insulin resistance also served as a crucial marker for conditions such as obesity, hypertension, dyslipidemia, and other metabolic syndromes (25, 26). In these metabolic diseases, the TyG index served an important predictive function.

Our research indicated a positive connection between the TyG and gallstone risk, with gallstone risk increasing by 60% as the TyG index rose. A one-unit increment in the TyG index led to a 40% increase in the gallstones risk. The underlying reason might be that insulin resistance was not only associated with metabolic syndrome but also closely related to the occurrence of gallstones (4, 11, 27, 28). Prior studies have demonstrated that insulin resistance, through the dysregulation of the transcription factor FOXO1 in hepatocytes, induced ABCG5 and ABCG8, thus enhancing biliary cholesterol secretion and disrupting cholesterol homeostasis in the bile duct, leading to cholesterol gallstone formation (29). As the TyG index was a reliable indicator of insulin resistance, an increase in the TyG index signified heightened insulin resistance, potentially resulting in an increased risk of gallstones. On the other hand, the gut microbiota might also play a significant role in this process. Earlier studies revealed that individuals with gallstones have decreased levels of Lachnospiraceae and Bacteroidales and increased levels of Ruminococcaceae and Spirochaetae in their intestinal flora. Notably, an increase in the abundance of Spirochaetae led to elevated levels of bile acids in the feces (30, 31). A study on the gut microbiota and insulin resistance showed that individuals with a higher number of Bacteroidales have a lower degree of insulin resistance (32). Thus, it was evident that the gut microbiota was linked to both insulin resistance and gallstones. Given that the TyG index was a reliable measure of insulin resistance, an elevation in the TyG index reflected an increase in insulin resistance, leading to alterations in the gut microbiota, notably Bacteroidales, and consequently increased the risk of gallstones. Prospective clinical research with large samples was necessary to further corroborate our findings.

The strengths of this study included, first, that the data came from the large sample NHANES database, which was representative and readily accessible. The findings based on this data were reliable and credible. Furthermore, confounding factors were extensively controlled for using multivariate, stratified, and interaction analyses. Lastly, fasting triglycerides and blood glucose, which made up the TyG index, were extensively utilized in clinical settings and were conveniently obtainable, thus supporting their broad clinical application.

It was undeniable that this study has some limitations. One limitation, inherent to the observational nature of the current research, was the inability to precisely ascertain the causal correlation between the TyG index and gallstone risk. This also pointed to the necessity for future prospective research to determine causality based on large sample data. Additionally, there were still some unknown biases, despite efforts to minimize them using various statistical methods. Finally, the diagnosis of gallstones was confirmed through questionnaire results, which might introduce some bias into the findings of the article.

## Conclusions

This was the first article to reveal a positive association between the TyG and gallstone risk, establishing an initial understanding of their relationship. This signified that as the TyG index progressively rose, gallstones risk enhanced accordingly. The potential cut-off value for the TyG index was 6.19. Above this threshold, every unit rise of the TyG was associated with a 40% higher risk of developing gallstones. Examining the continuous changes was essential in clinical settings because it aided doctors in more personalized assessments of individual risks for gallstones. Moreover, this study first demonstrated that non-linear relationship was not be found between the TyG and gallstones. Briefly, considering the convenience and ease of obtaining the TyG index in clinical practice, it held promising prospects for clinical application.

## Data availability statement

The original contributions presented in the study are included in the article/**Supplementary Material**. Further inquiries can be directed to the corresponding author.

## Ethics statement

The studies involving humans were approved by the Institutional Review Board at the Centers for Disease Control and Prevention. The studies were conducted in accordance with the local legislation and institutional requirements. The participants provided their written informed consent to participate in this study.

## Author contributions

QL: Conceptualization, Formal analysis, Methodology, Software, Visualization, Writing – original draft. YC: Conceptualization, Formal analysis, Methodology, Software, Visualization, Writing – original draft. QP: Data curation, Methodology, Software, Writing – original draft. CL: Conceptualization, Project administration, Supervision, Validation, Writing – review & editing.
