# Supplementary material for: Relationship between triglyceride-glucose index and gallstones risk: a population-based study
Source: Front Endocrinol (Lausanne). 2024 Jul 11;15:1420999. doi: 10.3389/fendo.2024.1420999 (PMC11269096; doi:10.3389/fendo.2024.1420999)
Supplement: Supplementary file 1 [file DataSheet_1.doc]

**Supplementary Online Content**

**Supplementary Methods**

Races were categorized into White people or Non-White people. Smoking status was grouped as never/former-smoker, or now-smoker, per their responses to whether they currently smoked and had spent at least 100 cigarettes in their life. Alcohol consumption was grouped by alcohol intake. The participants who consumed less than twelve drinks in a lifetime was categorized as a never drinker; those who consumed one to two drinks for female or one to two for male was categorized as a mild-moderate drinker; those who consumed more than two drinks for female and three drinks for male was categorized as a heavy drinker. Mild-moderate or heavy drinker was grouped as now-drinker. Having a history of hypertension is grouped as taking anti-hypertensive medication for hypertension or a mean systolic blood pressure (SBP) greater than or equal to 140 mmHg and/or a mean diastolic blood pressure (DBP) greater than or equal to 90 mmHg when SBP and DBP were collected at MEC or self-reporting of doctor's diagnosis of hypertension. Diabetes was grouped as receiving oral hypoglycemic agents or insulin, the hemoglobin concentrations were higher than or equal to 6.5% or fasting glucose higher than or equal to 7.0 mmol/l, or random fasting glucose higher than or equal to 11.1 mmol/l or 2-hour glucose tolerance glucose higher than or equal to 11.1 mmol/l or self-reported of a doctor's diagnosis of diabetes.
